# Primary Central Nervous System Lymphomatoid Granulomatosis: Systemic Review

**DOI:** 10.3389/fneur.2020.00901

**Published:** 2020-09-11

**Authors:** Yuanyuan Xiang, Cuicui Liu, Yuan Xue, Shan Li, Yanling Sui, Jifeng Li, Qinjian Sun, Xiaohui Liu

**Affiliations:** Department of Neurology, Shandong Provincial Hospital Affiliated to Shandong First Medical University, Jinan, China

**Keywords:** lymphomatoid granulomatosis, central nervous system, primary, imaging, neuropathology, Epstein-Barr virus

## Abstract

Lymphomatoid granulomatosis (LYG) is an infrequent lymphoproliferative disease that typically involves the lungs, but may also affect the central nervous system (CNS). Isolated CNS involvement is very rare, and its clinicopathological features have not been fully elucidated. Here, we systematically reviewed the English literature through PubMed to collect all relevant case reports and small case series with pathologically confirmed primary CNS-LYG. A total of 29 relevant articles with 40 cases were included in this systemic review. In cases where T cells and B cells were compared, T cells were predominant in 19 (79.2%), and B cells were predominant in 5 (20.8%). The overall infection rate of EBV was 48.1% (13/27), among which the infection rate was 40.9% (9/22) in immunocompetent patients and 80% (4/5) in immunodeficient (HIV-infected) patients. Among the patients who underwent pathological grading, 35.7% (5/14) were at grade I, 42.9% (6/14) were at grade II, and 21.4% were at grade III. In conclusion, primary CNS-LYG is closely related to EBV infection and some cases may be predominantly T-cell phenotype. Surgical resection may be effective for mass-like lesions, although there is still a lack of standard therapeutic regimen. Accurate grading of lesions is essential for treatment selection and prognosis evaluation.

## Introduction

Lymphomatoid granulomatosis (LYG) is a rare Epstein–Barr virus (EBV)-associated lymphoproliferative disease, characterized by B-cell proliferation of uncertain malignant potential accompanied by extensive reactive T-cell infiltrate (1–3). This disorder was first described by Liebow et al. (4). To date, its prevalence has not been accurately measured and the information is mainly from case reports and small case series. The most frequently involved site of LYG is the lung (90%), but it may also affect multiple extrapulmonary sites, including the skin (25–50%), the kidney (30–40%), the liver (29%), and the central nervous system (CNS) (26%) (4–7). In rare cases, patients with LYG initially present with neurological symptoms and lack of symptoms in the lungs and other regions, which may result in significant delays in diagnosis (8, 9). Since the isolated CNS involvement is very rare, its clinicopathological features have not been fully elucidated yet. We herein conduct a systematic literature review of primary CNS-LYG, focusing on summarizing its neuroimaging, histopathological features and the relationship with EBV infection.

## Methods

We used the following terms to search the PubMed database: “lymphomatoid granulomatosis,” “central nervous system,” “brain,” and “cerebral.” Among the patients reported, we selected the adults with pathological evidence of CNS involvement and excluded those with extra CNS involvement. We only evaluated the English literature, and the references of each pertinent study were also reviewed to search possible pertinent articles.

## Results

The last PubMed search was performed on January 1, 2019. A total of 29 relevant articles with 40 cases were included in this systemic review (Table 1). There were 26 males and 13 females, with a male/female ratio of 2.0, while in one case, gender was not reported. The mean age was 47.75 years (ranging from 19 to 74). Except for 3 patients who did not describe the follow-up, the average follow-up time of the remaining 37 patients was 27.3 months (ranging from 3 weeks to 221 months).

**Table 1:** Literature review of 40 cases of primary CNS-LYG.

**No**	**Publication year**	**Age/gender**	**Presentation**	**HIV**	**Localization**	**Type**	**Grading**	**EBER ISH**	**Diagnosis**	**Predominant lymphocyte at the lesion**	**Treatment**	**Response**	**Follow-up**
1	1984 (10)	51/M	Headache, confusion	No	Right temporal lobe, insula	Mass	nd	nd	Biopsy	nd	Surgery	Remission	14 months: disease-free
2	1984 (11)	41/M	Uncontrollable rapid breathing, seizure, confused, slow mentation	No	Bilateral hemispheres, greatest in frontal area	Diffuse	nd	nd	Biopsy	nd	Steroids, cyclophosphamide, RT	Remission	5 weeks: symptom-free
3	1987 (12)	36/F	Blurring of vision, headache, diplopia	No	Right temporal lobe, meningeal, cavernous sinus infiltrate	Mass	nd	nd	Biopsy	T-cell origin	Surgery, RT	Remission	14 months: symptom-free
4	1991 (13)	57/M	Headache, urinary stool incontinence, right hemiparesis	No	Left frontal lobe, corpus callosum	Mass	nd	nd	Biopsy	T cells, B cells	Surgery	nd	nd
5	1991 (14)	44/F	Personality change, depression, irritability	No	Cerebral, cerebellar, brain stem	Diffuse	nd	nd	Biopsy	nd	Steroids, cyclophosphamide	Improvement	7 months: relapsed
6	1993 (15)	45/M	Confusion, severe headache	Yes	Bilateral basal ganglia	Mass	nd	nd	Autopsy	Most T-cell origin	Steroids	Limited	3 weeks: died
7	1994 (16)	60/M	Incoordination, minor speech difficulties, headache, weight loss, vertigo	No	Right cerebellar hemisphere	Mass	nd	Positive	Biopsy	T cells, B cells	Surgery, RT	Remission, 16 months later deteriorated	18 months: died
8	1995 (17)	65/M	Headache, left-sided weakness	No	Right frontal, paratrigonal regions, left para ventricle	Mass	Nd	nd	Biopsy	T-cell predominant	Surgery, steroids, RT	Remission	40 months: symptom free.
9	1996 (18)	56/M	Seizures, drowsiness, headache, left-sided weakness	No	Right temporal lobe, later widespread infiltrate	Mass, diffuse	nd	nd	Biopsy	T-cell immunophenotype	RT, steroids	Deteriorated rapidly	More than 2 years: died
10	1996 (18)	39/M	Upper respiratory tract infection with ataxic paraparesis	No	Left pontomedullary region, later brainstem, cerebellum	Diffuse	nd	nd	Autopsy	T-cell immunophenotype	Steroids	Initially improved, finally deteriorate	11 months: died
11	1996 (18)	51/M	Allodynia, diplopia, dysgeusia, hemiataxia, unsteady gait, weight loss	No	Right cerebellar hemisphere	Mass	nd	nd	Biopsy	T-cell immunophenotype	Steroids, RT	Considerable improvement	5 years: disabled
12	1996 (18)	20/M	Vertigo, ataxia, weakness of the upper legs, “tremulousness”	No	Cerebellum, right lateral pons	Mass	nd	nd	Biopsy	Immunophenotypic studies insufficient	Refused specific treatment.	Continue deteriorated	12 months: died
13	2001 and 2007 (19, 20)	72/M	Spastic gait paraparesis	No	Bilateral cerebral white matter, cerebellum, brain stem	Mass, diffuse	nd	Negative	Biopsy	T-cell predominant	Steroids, RT	Remission but later relapsed	19 months: died
14	2001 and 2007 (19, 20)	58/M	Spastic gait paraparesis	No	Bilateral cerebral, cerebellar white matter, brain stem	Diffuse	nd	Negative	Biopsy	T-cell predominant	Steroids, RT	Remission	5 years: disease free
15	2001 and 2007(19, 20)	35/M	Spastic gait choreoathetosis, dementia, aniscoria, apraxia, rigidity, spasticity	No	Brain stem, bilateral cerebral white matter	Diffuse	nd	Negative	Biopsy	T-cell predominant	Steroids	Remission	42 months: died
16	2001 (21)	48/M	Headache, vertigo, gait ataxia, dysmetria	Yes	Left cerebellum	Mass	nd	Negative	Biopsy	T-cell predominant	Declined RT	Improvement	1 year: no progression
17	2006 (22)	44/M	Dementia, inactive, gait disturbance, headache	No	Bilateral frontal, parietal white matter	Diffuse	Grade 1	Negative	Biopsy	T-cell predominant	Methotrexate, CHOP chemotherapy	Gradually improved	nd
18	2006 (23)	56/M	Disturbance of consciousness, seizure	Yes	Left frontal lobe	Mass	Grade 1	Positive	Biopsy	T-cell predominant	RT	Deteriorate	2 months: alive
19	2007 (24)	37/?	Headache, dizziness, weakness	Yes	Basal ganglia	Mass	nd	Positive	Autopsy	T-cell predominant	None	Deteriorate	6 months: died
20	2008 (8)	55/M	Headache, memory disturbance, ataxia, a visual field defect	No	Cerebellum, occipital lobes, later multiple lesions	Mass, diffuse	Grade 1	Not performed Immunostaining negative	Biopsy	T-cell predominant	Surgery, steroids	Improved	3.5 years: no recurrence
21	2009 (25)	65/M	Visual disturbance, hemianopsia	No	Right occipital, temporal lobes	Mass	nd	Negative	Biopsy	T-cell predominant	Surgery, steroids	Effective	10 months: no recurrence
22	2009 (9)	38/F	Memory impairment, fever, malaise, left hemiparesis	No	Right basal ganglia, right frontal lobe, right temporal lobe	Mass	Grade 3	Negative	Biopsy	T cells, B cells	Steroids, methotrexate, RT	Effective	221 months: disease free
23	2009 (9)	38/M	Headache, vomiting, fever, impairment of consciousness	No	Fronto-temporo-insular lobes	Mass	Grade 1	Negative	Biopsy	T cells, B cells	Surgery, steroids	Effective	21 months: disease free
24	2009 (9)	52/F	Confusion, gait imbalance, urinary incontinence	No	Cortical–subcortical bifrontal lobe, corpus callosum	Mass	Grade 2	Negative	Biopsy	T cells, B cells	Steroids, cyclophosphamide therapy	Effective	24 months: disease free
25	2009 (9)	49/M	Paraparesis, legs paresthesias	No	Subcortical occipital lobe, cervical spinal cord, leptomeninges	Mass, diffuse	Grade 1	Negative	Biopsy	T cells, B cells	Steroids	Effective	18 months: disease free
26	2010 (26)	44/F	Confusion, memory loss, aphasia, seizures, dysphagia, hemiplegia	Yes	Periventricle, left frontal lobe	Mass, diffuse	nd	Positive	Biopsy	T-cell predominant	Steroids	No benefit	9 months: died
27	2010 (27)	21/M	Seizure, headache, dizziness, numbness	No	Left parietal lobe	Mass	Grade 2	nd	Biopsy	B-cell phenotype	Surgery, steroid, RT	Partial remission	13 months: no recurrence
28	2010 (27)	19/F	Weak muscles, headache, vomiting	No	Left frontal, posterior parietal cortex	Mass	Grade 2	nd	Biopsy	B-cell phenotype	Surgery, chemotherapy	Fully recovered.	15 months: no recurrence
29	2011 (28)	36/F	Vision loss, amaurosis, exophthalmos, pain, paresthesia	No	Cavernous sinus, orbital apex, left temporal lobe	Mass	nd	Positive	Biopsy	B cells, T cells	Surgery, chemotherapy, RT	Good recovery	4 years: disease free
30	2011 (28)	73/F	Left hemiparesis	No	Right posterior temporal lobe	Mass	nd	Positive	Biopsy	B cells, T cells	Steroids, surgery	Good recovery	5 years: no recurrence
31	2011 (28)	54/M	Vision loss, exophthalmos, pain, paresthesia.	No	Cavernous sinus, orbital apex	Mass	nd	Negative	Biopsy	B cells, T cells	Steroids, surgery	Good recovery	3 years: disease free
32	2011 (29)	74/F	Headache, disoriented, hemianopsia	No	Left medial temporal lobe	Mass	Grade 3	Positive	Biopsy	T cells, B cells	Surgery	Deteriorated	14 months: died
33	2013 (7)	67/M	Visual impairment, hemianopsia	No	Suprasellar region	Mass	Grade 2	Positive	Biopsy	T-cell predominant	Surgery, steroids, methotrexate, RT	Recovered fully	12 months: no recurrence
34	2015 (30)	53/M	Cognitive decline, extrapyramidal features, altered sensorium with seizures	No	Subependymal, ependymal regions, anterior/basal regions, brain stem, spinal cord	Diffuse	nd	nd (EBV LMP-1: negative)	Biopsy	T cells, B cells	Cyclophosphamide, steroids	Effective	3 months: partial remission
35	2017 (31)	49/M	Aphasia	Yes	Multiple lesions, mainly in left parietal, temporal lobes	Mass, diffuse	nd	Negative	Biopsy	T-cell predominant	Anti-retroviral therapy	Complete remission	18 months: no recurrence
36	2017 (32)	51/F	Ataxia, dizziness, facial palsy, hearing impairment, weakness	No	Brachium pontis, occipital lobe cerebellum, para-lateral ventricle	Mass	Grade 2	Positive	Biopsy	T-cell predominant	Steroids, azathioprine	Improved quickly but not completely	5 months: died
37	2018 (33)	31/F	Numbness, diplopia, nausea, dizziness	No	Brainstem, cerebellum, later all over the brain	Mass, diffuse	nd	Negative	Biopsy	B-cell predominant	Steroids, cyclophosphamide	Gradually less responsive	46 months: died
38	2018 (34)	62/F	Headache, impairment of cognitive function and movement	No	Parietal lobe, thalamus, later bilateral cerebral hemispheres	Mass, diffuse	Grade 3	Positive	Biopsy	B-cell predominant	Steroids, continuing IS	Markedly improved	29 months: no recurrence
39	2018 (34)	42/F	Headache, muscle weakness	No	Left paracentral lobule, corpus callosum	Mass	Grade 2	Positive	Biopsy	B-cell predominant	Steroid, continuing IS	Markedly improved	23 months: no recurrence
40	2018 (35)	22/M	nd	No	Right frontotemporal region	Mass	nd	Positive	Biopsy	nd	Not opted for treatment	nd	nd

*LMP-1, latent membrane protein 1; RT, radiation therapy; IS, immunosuppressive treatment; nd, not described*.

In our review, except for 1 case that did not describe the main symptoms, 17 patients had headaches (43.6%) in the remaining 39 patients. Of 40 patients, 25 had supratentorial lesions, 5 patients had infratentorial lesions, while the remaining 10 patients had lesions both above and below the tentorium or in the spinal cord. Twenty-five patients (62.5%) manifested as mass-like lesions, 7 patients (17.5%) presented as diffuse infiltrating changes, while in 8 cases (20%), there were both space-occupying and infiltrating lesions.

Except for 5 cases that did not describe T cells and B cells in the brain lesions, T cells were predominant in 19 cases, B cells were predominant in 5 cases, and 11 cases described both T-cell and B-cell infiltrates, but did not compare them in the remaining 35 cases.

In our review of the 27 patients who described the details of EBV infection, 24 were detected by *in situ* hybridization of EBV-encoded RNA (EBER ISH), 1 was detected by EBV latent membrane protein 1 (LMP-1), 1 was detected by both EBER ISH and EBV-PCR, and 1 was detected by immunostaining. Of these 27 patients, 12 were positive for EBER ISH in brain biopsy or autopsy, 1 was positive for CSF EBV-PCR but negative for EBER ISH in brain biopsy a month later, and 14 were negative. Therefore, the positive rate of EBV infection was 48.1% in our review of CNS-LYG.

Six patients were found to combine with HIV infection, 3 of whom were positive for EBER ISH, 2 were negative, and 1 did not describe the details of EBV infection. One of the EBER ISH negative patients was positive for EBV-PCR in CSF a month before and the other EBER ISH negative patient was infected with HIV-2.

Fourteen patients underwent pathological grading, including 5 patients at grade I, 6 patients at grade II, and 3 patients at grade III.

Fifteen patients underwent surgical resection and 14 of them had mass-like lesions, while 1 patient manifested as both mass-like and diffuse infiltrating changes. After surgical resection (grade III), the symptoms of one patient continued to deteriorate, and later died after giving up treatment, and in another patient, complete remission lasting 16 months after treating with surgery and radiotherapy before the lesion transformed into a lymphoma, while in a third patient, the effect was not described. Of the remaining 12 patients who underwent surgery, 1 patient was relieved only by surgical resection, and 11 patients underwent surgery combined with steroids, chemotherapy, or radiotherapy, and achieved partial or complete remission during follow-up without recurrence.

Twelve patients died during follow-up, and among the three patients who underwent autopsy, two patients completed biopsy while alive. Among the 25 surviving patients, 1 patient experienced symptom deterioration, 1 patient relapsed, and 1 patient was severely disabled during follow-up; the remaining 22 patients achieved partial or complete remission after treatment, with good quality of life and no recurrence. Of the six HIV-infected patients, one deteriorated and three died during follow-up, while the other two patients had a better prognosis, including one with HIV-2 and the other with complete remission after first-line anti-retroviral therapy.

## Discussion

LYG is a rare EBV-associated, T-cell rich, B-cell lymphoproliferative systemic disease (systemic-LYG) (3), which is usually a male preponderance and generally peaks between the fifth to sixth decades (36). CNS involvement occurs in approximately 30% of patients (19), and it is a serious prognostic factor, with a mortality rate of 86% compared with 66% for patients without CNS after 14 months (5).

Isolated CNS involvement has been rarely reported and the diagnosis of primary CNS-LYG relies mainly upon biopsy and lacks other specific tests. So far, we have only found 40 cases of primary CNS-LYG in the English literature (7–35) (Table 1).

### Clinical Presentation and Neuroimaging

Various clinical symptoms, such as headache, seizure, diplopia, hemiplegia, altered consciousness, Parkinsonism, dementia, etc. (6, 37), may occur in primary CNS-LYG depending on the site involved, and primary CNS-LYG may involve any part of the CNS, such as the cerebrum, cerebellum, basal ganglia, brainstem, etc., without specificity. In our review, headaches were the most common symptoms and supratentorial involvement was more common.

The brain lesions of LYG mainly manifest as mass-like or diffuse infiltrating changes (38), which may be single or multiple. Primary CNS-LYG might more frequently manifest as intracranial mass-like lesions than CNS involvement of systemic LYG (9). Mass-like lesions were more common than diffuse infiltrating changes in the present systematic review of primary CNS-LYG.

Although not specific, MRI examination is indispensable for detecting intracranial lesions and monitoring changes during follow-up (39). The multiple punctate, linear, and ringlike enhancement can be seen on Gadolinium-enhanced T1-weighted imaging of primary CNS-LYG patients, which is likely to represent the pathological features of involving the vessel wall and perivascular tissue (19, 30).

Nishihara and coworkers pointed out in their case report that in the same lesion site, FDG-PET scan showed a low uptake indicating necrotic lesions, while methionine (MET)-PET scan showed a high uptake indicating hypercellular proliferative lesions (25). This mismatch accumulation was considered to be characteristic of LYG (25). In addition to PET-CT, other advanced imaging techniques, such as MRS and T2^*^-weighted MRI have also been applied to the study of LYG. However, the diagnostic value of these techniques for primary CNS-LYG still needs further study.

### Neuropathology

LYG is characterized by an angiocentric and angiodestructive infiltrate of lymphocytes, histiocytes and occasional plasma cells. Inflammatory cells, such as neutrophils and eosinophils, usually do not exist. Necrosis is variable and more pronounced in higher-grade lesions. Well-formed granulomas do not exist (6). “Lymphomatoid granulomatosis” is named because its clinical manifestations are similar to granulomatous polyangiitis and its histological features are similar to lymphoma. The term “granulomatosis” is not the actual granulomatous inflammation (40).

Whereas, a predominant B-cell phenotype of systemic LYG is generally accepted, the results of this review support the idea that some cases of primary CNS-LYG may be predominantly T-cell phenotype. All the HIV-infected patients reviewed showed T-cell dominant angiocentric infiltrate in brain tissue histopathological examination (15, 21, 23, 24, 26, 31). Nishihara et al. analyzed T-cell receptor (TcR) and immunoglobulin heavy chain (IgH) gene rearrangements in their cases of CNS-LYG and proposed that a large part of CNS-LYG could be classified as a low-grade malignant lymphoproliferative disease with essential T-cell phenotype (20). However, primary CNS-LYG is still a controversial disease, and its exact immunophenotype needs further study.

According to the WHO classification, LYG grading is based on the proportion of large lymphoid cells, the number of EBV-positive B cells per high-power field and the amount of necrosis (41). About 50% of grade II and 70% of grade III LYG present as clonal immunoglobulin gene rearrangements, but grade I lesion rearrangements are not common (42).

### Differential Diagnosis

Due to the presence of many mimickers, primary CNS-LYG is diagnostically challenging (43–46), and should be differentiated from both space-occupying and diffuse cerebral lesions (30), such as primary CNS lymphoma, vasculitis, Wegener's granulomatosis, malignant glioma, metastatic tumors, chronic lymphocytic inflammation with pontine perivascular enhancement responsive to steroid (CLIPPERS), multiple sclerosis, etc. Primary CNS lymphoma is an important differential diagnosis of LYG grade III; however, it lacks the pattern of vascular invasion/destruction seen in primary CNS-LYG, although with a unique histopathological feature of angiocentric growth. In addition, some patients of LYG grade III may still have spontaneous remission with the change of immune status (47). Both vasculitis and primary CNS-LYG manifest as vascular damage caused by polymorphic infiltrate of the vessel wall, resulting in infarct-like tissue necrosis. The significant angiocentric lymphoid infiltrate, evidence of EBV-positive cells and lack of eosinophils or giant cells suggest a diagnosis of primary CNS-LYG, whereas the presence of granulocytes, eosinophils, and microabscess suggests vasculitis.

### EBV Expression

EBV is associated with a series of lymphoproliferative disorders. LYG is a rare EBV-driven B-cell lymphoproliferative process known for its angiocentricity and angioinvasiveness (48), and it is speculated that these patients have EBV immune surveillance disorders. In LYG, organ damage may be partly mediated by the host's immune response to EBV, while the pathological damage in most other EBV-driven B-cell lymphoproliferative diseases is caused by the direct expansion of EBV-infected B cells (49). Post-transplantation lymphoproliferative disease (PTLD) is an EBV-related B-cell lymphoproliferative process presenting as polymorphous or monomorphous, and shares many clinical features with LYG. In most cases, PTLD can be differentiated from LYG by the lack of a background of angiocentric T-cell infiltrate, the presence of prominent plasmacytic differentiation, and elevated median viral loads (42). Currently, the number of EBV-positive B cells per high-power field is used as part of the LYG grading criteria in the 2008 WHO classification (41), and grading is essential for guiding treatment and assessing prognosis.

Lucantoni et al. (9) reported that unlike systemic LYG, primary CNS-LYG appears not to be associated with EBV, while our review has shown that the positive rate of EBV infection was 40.9% (9/22) for immunocompetent patients, 80% (4/5) for immunodeficient (HIV-infected) patients, with a ratio of ~1:2. Thus, we speculate that the association of EBV with primary CNS-LYG is not restricted to patients with severe immunodeficiency and EBV may also be implicated in immunocompetent patients. However, it is still unclear whether EBV actually causes the disease or it is only a reflection of secondary activation, so large series of cases are needed to clarify this issue. AIDS-related primary CNS-LYG may be triggered by reactivation of EBV in the CNS under immunosuppressive conditions, followed by active T-cell infiltrates (31). However, it remains unknown whether these immunocompetent patients have other occult immunodeficiency. In addition, since treatment options and follow-up times were varied, we were unable to analyze the effect of EBV on survival time.

### Therapy and Prognosis

No standard therapeutic regimen has been established for primary CNS-LYG, and alternative treatment options include surgical resection, steroids, chemotherapy, radiation therapy, and anti-retroviral therapy for HIV patients. In our review, surgical resection may be effective for mass-like lesions.

Regarding the prognosis of primary CNS-LYG, it seems to be correlated with a better life expectancy than systemic-LYG with CNS involvement (9) and is closely associated with grading. In the present review, 1 patient developed lymphoma 16 months after complete remission and then died 2 months later (16). The biopsy of another patient showed LYG and focal conversion to lymphoma (11). However, neither case was graded, because pathological grading was not achieved in the 1980s and 1990s. We speculate that the most likely pathological grade for the two patients was grade III, for LYG grade III lesion is considered a subtype of diffuse large B-cell lymphoma (47).

## Conclusion

Primary CNS-LYG is rarely reported, and its diagnosis relies mainly upon biopsy and differential diagnosis should consider diffuse and mass-like cerebral lesions. MRI examination is crucial for detecting intracranial lesions and monitoring changes during follow-up, although not specific. Unlike systemic LYG, some cases of primary CNS-LYG may be predominantly T-cell phenotype. Primary CNS-LYG is also closely related to EBV infection, which is not only in patients with severe immunodeficiency but also in immunocompetent patients. So far, there is no standard therapeutic regimen established for primary CNS-LYG and surgical resection may be effective for mass-like lesions. Accurate lesion grading is crucial for selecting the appropriate treatment and assessing prognosis.

## Author Contributions

XL and YXi conceived the study, performed literature research, and wrote the paper. CL, YXu, SL, and YS helped to perform literature research. XL, JL, and QS revised the paper. All authors contributed to the article and approved the submitted version.

## Conflict of Interest

The authors declare that the research was conducted in the absence of any commercial or financial relationships that could be construed as a potential conflict of interest.
